# Baltimore College of Dental Surgery

**Published:** 1846-09

**Authors:** 


*Baltimore College of Dental Surgery.*—It will be gratifying to the friends of this institution to know, and we take pleasure in announcing the fact, that all the chairs are now filled. A. Westcott, M. D., of Syracuse, New York, has been electedto the Chair of Operative and Mechanical Dentistry.

We further announce that Dr. Cyrenius O. Cone has been appointed Demonstrator of Mechanical Dentistry. This appointment, made after due deliberation, also evinces sound judgment and discrimination. We know of none better qualified to fill this situation creditably than the gentleman upon whom the choice has fallen.

The faculty will be able to welcome their class in a new and convenient building, located in the very centre of the city, but a few rods from the Battle Monument. Great care has been taken to make it suitable for the purpose to which it will be devoted. The Museum has also been enlarged by the addition of valuable models, &c. Upon the whole, the college will commence its next session under highly favorable auspices.

*Balt. Ed.*

